# Telehealth group intervention for adolescents and young adults with cancer: a feasibility pilot study protocol

**DOI:** 10.1186/s40814-024-01590-5

**Published:** 2025-01-31

**Authors:** Sherilynn F. Chan, Joanna Patten, Nancy Lau

**Affiliations:** 1 Department of Psychiatry and Behavioral Sciences, University of Washington, Seattle, WA USA; 2 Division of Psychiatry and Behavioral Medicine, Seattle Children’s Hospital, Seattle, WA USA; 3 Center for Child Health, Behavior and Development, Seattle Children’s Research Institute, Seattle, WA USA

**Keywords:** Adolescents and young adults, Cancer, Telehealth, Social support

## Abstract

**Background:**

Providing opportunities for social interaction and access to psychosocial interventions are 2 of the 15 Standards for Psychosocial Care for Children with Cancer and Their Families. Peer relationships are especially important among adolescents and young adults (AYAs), and cancer disrupts this aspect of social development. Many AYAs with cancer report a desire to engage in peer support groups; however, this remains a critical unmet need. Telehealth is a cost-effective and increasingly common modality for delivering health care that reduces access barriers and may be an especially good fit for AYAs with cancer. Little work has evaluated the feasibility and acceptability of peer support groups for AYAs with cancer. The purpose of this study is to evaluate the feasibility and acceptability of a telehealth peer support group intervention for AYAs with cancer.

**Methods:**

Telehealth group interventions are offered twice yearly as standard of care at Seattle Children’s Hospital to AYAs on or off treatment for cancer or a brain tumor. Group members are assigned to a High School Group (grades 9–12) or an AYA Group (spring of grade 12 or older). Aim 1 is to determine the feasibility and acceptability of the intervention for all patients who participate in groups in their clinical care. Feasibility will be assessed based on a priori metrics (enrollment, attendance, attrition) for all group members. Group and telehealth acceptability will be assessed by a 16-item internally developed measure that was adapted from the Client Satisfaction Questionnaire. Aim 2 is to conduct patient stakeholder semi-structured interviews post-intervention with 20 AYAs to gather feedback and inform intervention refinement. Participants who opt-in for study procedures will also complete exploratory measures of social connectedness/isolation, depression, and benefit finding, pre- and post-intervention (Aim 3).

**Discussion:**

Findings from this pilot study will inform intervention refinement, intervention implementation, and sample sizes for future powered trials. This study will provide preliminary empirical evidence to support the development of effective group interventions for AYAs with cancer that increase opportunities for social interaction and access to peer support, with the potential to contribute to improved psychosocial care of AYAs with cancer.

## Background

Adolescents and young adults (AYAs) with cancer are an important yet understudied group with unique psychosocial needs [1]. Peer relationships are especially important among AYAs, and cancer disrupts this aspect of social development [2, 3]. AYAs with cancer miss out on developmentally important life experiences which impacts their opportunities for socialization and ability to develop and maintain peer relationships. AYAs with cancer report feeling socially isolated and different from their healthy peers [4, 5]. The impact of social isolation on mental health outcomes, ability to cope, and quality of life has been well-documented among AYA cancer patients and survivors [2, 4, 6–8]. Additionally, the COVID-19 pandemic has maintained and exacerbated experiences of social isolation among AYAs with cancer and survivors [9].

Adolescents and young adults report a strong desire for social interaction and peer support to promote coping with cancer treatment and survivorship [2, 4, 10]. In particular, AYAs express a desire to meet other patients and for greater access to cancer support groups and peers with shared illness experiences [1, 4, 11]. They identify other AYAs with cancer as important supports that can relate to and understand them in ways that people without a history of cancer cannot [11, 12]. The development and testing of evidence-based interventions to foster positive peer relationships between AYAs with cancer remain a critical unmet need [1, 4, 10].

Providing opportunities for social interaction and access to psychosocial interventions are 2 of the 15 Standards for Psychosocial Care for Children with Cancer and Their Families with moderate to strong evidence [4, 13]. In a survey of pediatric oncology treatment programs in the United States, only 38.9% of programs reported provision of support groups as a way to deliver care consistent with the psychosocial standard for social interaction [14]. Due to variability in the provision of psychosocial care both across and within pediatric oncology centers and increased calls for models of care that are cost-effective and outcome driven, efforts to standardize approaches and evaluate efficacy of clinical programs are becoming a priority [15]. The matrix and guidelines were developed to guide implementation of the standards with the potential to encourage greater uniformity of optimal psychosocial care across institutions [16]. The guidelines recognize that programs attempting to implement the Standards face various challenges, primarily related to funding, staffing, and billing/reimbursement [14, 17]. Furthermore, provision of peer support groups among patients in clinical care settings has many barriers, including geography, cost, and broad age range of participants [18].

It is imperative to develop and evaluate peer support group interventions that are tailored to AYAs’ specific developmental needs, improve access to care, and are sustainable, cost-effective, and outcome driven. Innovative approaches are needed to facilitate connections between AYA patients and survivors and meet this Psychosocial Standard of Care [19]. Telehealth is a cost-effective and increasingly common modality for delivering health care that reduces access barriers [20, 21]. Early evidence supports the feasibility of telehealth group interventions for AYAs with chronic illness, though few studies have examined efficacy and current findings are mixed [22]. An online, group-based, cognitive-behavioral therapy intervention for AYA cancer survivors ages 15–25 years (“Recapture Life”) was evaluated in a three-arm randomized controlled trial [23]. The intervention was compared to an online peer support group and a waitlist control. Immediately post-intervention, there were no significant group differences in quality of life or emotional distress. However, at 6-month follow-up, Recapture Life participants reported greater use of cognitive behavioral strategies compared to patients in the peer support group control. Additionally, therapeutic alliance and group cohesion were rated as high throughout the program [24]. In another recent study, an online cognitive-existential group psychotherapy program for AYAs with cancer ages 16–29 years was developed that consists of 8 weekly 90-min sessions [25]. This intervention combines skills from cognitive behavioral and third-wave therapies with existential approaches such as supportive-expressive group therapy. Sessions focus on an AYA-specific topic and include a combination of psychoeducation, practice of a therapeutic activity, and facilitated discussion. Findings provided evidence for the feasibility and acceptability of the intervention, and participants (*n* = 33) reported significant improvements in quality of life from pre- to post-intervention. Of note, participants reported that the most helpful aspects of the intervention were meeting, sharing experiences, and expressing feelings about personal experiences with other AYAs with cancer.

Thus, limited but growing evidence suggests that telehealth group interventions for AYAs with cancer are feasible and have a signal towards efficacy. A virtual mode of delivery of care may be ideal for AYAs with cancer as it overcomes several barriers. AYAs with cancer may have difficulty accessing care and peer connections in-person due to distance from the hospital or treating center, lack of transportation, and illness- and treatment-related factors, such as immunocompromised status, fatigue, pain, mobility issues, and other physical symptoms and limitations. However, it is not yet known if telehealth is a sustainable and cost-effective alternative to traditional in-person care. An additional important gap in the literature is the development and evaluation of a telehealth peer support group intervention that intentionally and primarily focuses on facilitating opportunities for social connection and sharing of illness and treatment experiences, rather than formal psychotherapy and skills teaching [4, 25]. Rigorous testing of such a telehealth peer support group intervention for AYAs with cancer is needed to support implementation of the psychosocial standard of care for social interaction. Empirical data are necessary to develop and inform evidence-based group interventions that can be standardized and disseminated across multiple sites.

To address this important gap in the literature, this mixed-methods study has two primary aims. Aim 1 is to determine the feasibility and acceptability of conducting an existing telehealth peer support group intervention for AYAs with cancer in a pilot study. This unique intervention focuses on facilitating social connections and peer support to reduce social isolation, emphasizes sharing of illness and treatment experiences, includes a narrative therapeutic activity, and flexibly prioritizes discussion topics based on group members’ preferences. We hypothesize that the group intervention will be feasible and acceptable based on a priori metrics (i.e., enrollment, attendance, attrition) and survey data (i.e., acceptability questionnaire). Aim 2 is to conduct patient stakeholder interviews to gather feedback on the existing group intervention and inform intervention refinement. We anticipate that AYAs will offer valuable feedback on perceived benefits, barriers/challenges, and suggestions for improvement via qualitative interviews. A third exploratory aim is to collect pre- post-intervention data on patient-reported outcomes, specifically self-reported social isolation/social connectedness, depressive symptoms, and benefit finding. Findings from this study will inform intervention refinement and adaptation for further testing and broader implementation. This article describes the protocol of this pilot study.

## Methods

### Setting and sample

We plan to recruit 20 participants who attend telehealth peer support groups at Seattle Children’s Hospital (SCH) in Fall 2023 and Spring 2024. If more than 20 AYA patients are eligible and express interest in research participation, we will select participants to ensure a representative and diverse sample. Our target sample size was determined based on empirical guidance on sample sizes for saturation in qualitative research [26] and group sizes that have ranged from 6 to 10 in prior years. Consistent with prior groups, it is expected that participants will represent a range of cancer and brain tumor diagnoses and include patients both on and off treatment. Participants’ race and ethnicity are expected to reflect that of the larger patient population served in the Cancer and Blood Disorders Center at SCH. The projected gender, racial, and ethnic composition represents the demographics of Washington state. It is expected that the majority (up to 70%) of AYA participants will be White, ~ 12% Hispanic/Latino, ~ 4% Black/African American, and ~ 8% Asian. Approximate sex composition based on prior enrollment rates from the Cancer and Blood Disorders Center outpatient clinic at SCH is 43% female and 57% male.

Inclusion criteria for group members are as follows: (1) AYA patients in ninth grade or older who have received treatment for cancer or a brain tumor or who have received a hematopoietic stem cell transplant (HSCT), (2) English speaking, and (3) physically located in WA state when participating in the telehealth group intervention due to licensing and billing constraints. There are no exclusion criteria for group. Referring providers consider the patient’s desire for social connection, stage of treatment, and potential impact on attendance, as well as the patient’s desire to actively or passively participate in group discussions. Data abstracted from the medical record for all AYAs who attend group will be used to address Aim 1. We received IRB approval for waiver of consent for Aim 1 data collection. Group members are eligible to participate in additional study procedures (addressing Aims 2 and 3) if they meet the following inclusion criteria for research participation: (1) enrollment in the telehealth peer support group intervention at SCH and (2) able to complete questionnaires and interview in English. Exclusion criteria for research participants who are enrolled in groups completing study procedures addressing Aims 2 and 3 are cognitive impairments that would limit ability to provide informed assent/consent, complete questionnaires, and/or complete interview, as determined by medical and/or psychosocial team members familiar with the patient.

### Overview of group intervention

The High School Cancer Group and AYA Cancer Group are available as standard of care to AYAs treated in the Cancer and Blood Disorders Center at SCH and were not adapted for the purposes of this study. These telehealth group interventions are offered twice a year (fall: September–October; spring: May–June) for AYAs on or off treatment for cancer, a brain tumor, or a medical condition requiring a HSCT. Each group meets for 6–8 weekly 1-h sessions via HIPAA-compliant Zoom and is led by 1–2 behavioral health providers (e.g., pediatric psychologist, master’s-level mental health therapist). The group functions to facilitate social connectedness, a sense of community, and psychological well-being among AYAs with cancer. Group members are assigned to a High School Group (grades 9–12) or an AYA Group (spring of grade 12 or older). Group curriculum and facilitation are adapted for developmental age. For example, there is more facilitation in the High School Group and less facilitation in the AYA group which is more self-directed.

The AYA Cancer Group was first developed in 2016 by a pediatric psychologist who observed that their individual therapy caseload of AYAs with cancer experienced similar feelings of loneliness, disconnection from their peers, and profound distress related to changes to facets of their identity. The original purposes of the group were to provide social connection, peer validation, and skill building. The initial group curriculum included structured skill instruction, drawing from evidence-based group skills training interventions for youth with emotional distress (e.g., distress tolerance skills, mindfulness skills from dialectical behavioral therapy) [27, 28]. However, group members provided feedback that a highly structured curriculum with skill instruction was less preferred. Instead, group members expressed preference for opportunities for social connection, sharing illness and treatment experiences, reflecting on the impact of cancer and treatment on developmentally salient themes (e.g., dating, moving out of the house), and peer validation. The AYA Cancer Group underwent several years of iterative adaptation in response to the expressed needs of group members. The High School Cancer Group was started in 2021 and functions similarly to the AYA Cancer Group but requires slightly more facilitation by group leaders.

Both AYA and High School Cancer Groups now follow a similar sequence with regard to content and process. In the first session, the group leader facilitates introductions among group members, which includes name, pronouns, patient location (e.g., city), and brief summary of cancer diagnosis and treatment history. These introductions are repeated if any new members join during a later group session. The facilitator then reviews group rules (e.g., confidentiality, inclusivity, respect for different perspectives and opinions) and provides an orientation to the purposes of group, including social connection and an opportunity for group members to give and receive support around illness and treatment-related experiences. Finally, the group facilitator introduces and normalizes a wide range of developmentally relevant topics related to cancer that have been included and addressed during previous groups. These topics include mental health, social support, identity, relationships, school, work, recurrence fears, grief/loss, fertility, and short- and long-term health effects of cancer treatment. Group members then select topics of interest and relevance from this list and/or add additional topics for further discussion at subsequent sessions. Sometimes group members reference this list, or the group facilitator draws from this list at subsequent sessions to support discussion.

Sessions 2 and 3 typically include review of introductions and narrative activities, during which group members discuss cancer- and treatment-related experiences in greater detail (further described below). Often, group members express a desire to discuss with their peers a cancer-specific experience that occurred recently or that they anticipate in the near future (e.g., cancer anniversary). Subsequent group sessions typically include more discussion of AYA-relevant discussion topics. The beginning of each group session typically includes members sharing their response to an ice-breaker question. As group members become more comfortable with one another, the beginning question can also include a more personal reflection question (e.g., What is going on with you this week?). At the end of each session, group members share something that they are anticipating for the next week as well as ways in which they can feel supported by other group members. For example, group members often share if they have upcoming scans or high school graduation or a job interview, and group members offer thoughts, encouragement, and well-wishes.

Group members often identify the need to practice disclosure of their diagnosis and treatment history in different social contexts and report varying levels of distress in doing so. A narrative therapeutic activity is facilitated throughout group, often during sessions 2 and 3, which involves group members describing their diagnosis and treatment history in greater detail and with more brevity. This activity is a key component of the group intervention and has three intended functions. First, it provides exposure and an opportunity for habituation for AYAs with greater or persistent distress in recalling their own treatment experiences and hearing others’ experiences. Second, it provides AYAs with opportunities to practice including varying levels of detail, which can be appropriate to different situations, and practice answering questions from other group members. This may highlight areas where AYAs may not understand parts of their diagnosis or treatment and therefore can lead to increased knowledge of their own health information and self-efficacy. Third, this narrative activity allows identification of unique and shared treatment experiences and emotional responses between group members, reducing isolation and facilitating social connection and group cohesion.

There is currently no maximum number of patients allowed; the highest number of patients enrolled in a group in prior years has been 18. Group members are welcome to attend as many of the sessions as they are able, acknowledging that medical treatment and symptoms can intermittently interfere with group attendance. Intentional efforts are made within the group to foster an inclusive environment, including reflecting on how inclusive and supportive the groups have been in the past, recognizing that group members have different experiences, treatment impacts, and intersecting identities. Group leaders encourage patients to contact the group leaders directly if any of them experience issues with group dynamics, comments, or content that contribute to a sense of exclusion. Group leaders also acknowledge that group members may connect outside of group intentionally or spontaneously at medical appointments or events related to cancer treatment (e.g., camps, fundraisers) and encourage inclusivity and respect for confidentiality outside of group. We have learned from prior groups, especially in the AYA group, that many group members have maintained contact with one another and have offered additional levels of support, including attending medical appointments with each other, meeting together when group is not in session, and sharing additional resources related to social connection (e.g., summer camps, volunteer opportunities).

### Procedures

All procedures are approved by the institutional review board (IRB) at SCH. Any protocol amendments will be approved by the IRB prior to implementing any procedural changes.

#### Recruitment and informed consent

After enrollment in either the High School Cancer Group or AYA Cancer Group and prior to the first session of group attended, potential subjects/their caregivers will be contacted by phone by a clinical research coordinator to explain the opt-in study procedures for Aims 2 and 3, invite participation, and discuss informed consent. An overview of the study and potential risks and benefits will be described to patients/caregivers. If patients have an active MyChart account, potential participants and their caregivers may also receive a recruitment message through MyChart informing them about the study. Eligible AYAs who agree to participate (and caregivers when applicable) will view and digitally sign an electronic information sheet via the REDCap database using the REDCap e-consent framework to indicate their understanding and willingness to participate. All patients over 18 will provide signed informed consent prior to study participation. Patients ages 17 and younger will provide signed assent, and their designated caregiver or guardian will provide signed informed consent. A waiver of consent was approved for Aim 1 data collection via medical chart abstraction.

#### Data collection

The number of referrals to group, number of patients who enroll in group, and number of sessions attended by each group member will be abstracted from the electronic medical record (Epic). Reasons for declining (i.e., not enrolling in group after a referral has been placed) will be noted by the scheduling team in Epic. During the last group session, all group members complete a post-intervention internally developed acceptability questionnaire via REDCap as part of routine measurement-based care at SCH. Group members who do not attend the last group session are sent the questionnaire electronically to complete as soon as possible and within 3-month post-group. Clinical and demographic data for group members will be abstracted from the medical record to determine group member characteristics. These procedures will be conducted to address study Aim 1 and will involve all group members.

Participants who consent to this study will also complete a demographic/background questionnaire at baseline, the Revised UCLA Loneliness Scale, PROMIS-depression scale, and Benefit Finding/Burden Scale for Children at baseline and post-intervention, and an individual semi-structured qualitative interview post-intervention. All questionnaires will be administered via REDCap. The baseline timepoint window will be as early as 2–4 weeks before group begins and up until prior to the first group session the participant attends. Post-intervention questionnaires will be completed within 3 months following group and as close to the end of group as possible. Email reminders will be sent via REDCap, and clinical research coordinators will call participants to remind them to complete questionnaires as needed. To minimize missing data, a study team member will review measures for completeness and follow up with participants if there are items or measures that they skipped. Within 3 months following group, phone or virtual (e.g., Zoom, Microsoft Teams) interviews will be scheduled with a clinical research coordinator. Semi-structured interviews will be audio-recorded, transcribed verbatim, and de-identified. See Table 1 for schedule of enrollment, intervention, and data collection.

**Table 1:** Schedule of enrollment, group intervention, and data collection

	*Within 2–4 weeks prior to group start and prior to the first group session attended*	*Group intervention (6–8 weeks)*	*Within 3-month post-group*
**Study enrollment** 1. Study recruitment after patient enrolls in group intervention 2. Informed consent	X		
**Baseline measures** 1. Demographic/background questionnaire 2. Revised UCLA Loneliness Scale 3. *PROMIS* Emotional Distress — Depression Short Form (ages 18 + years or *PROMIS* Pediatric Depression Short Form (ages < 18 years) 4. Benefit Finding/Burden Scale for Children	X		
**Participation in group intervention as standard clinical care**		X	
**Post-group measures** 1. Acceptability questionnaire 2. Revised UCLA Loneliness Scale 3. *PROMIS* Emotional Distress — Depression Short Form (ages 18 + years or *PROMIS* Pediatric Depression Short Form (ages < 18 years) 4. Benefit Finding/Burden Scale for Children			X
**Semi-structured qualitative interview**			X

#### Participant incentives

Participants will receive an electronic gift card by email following completion of the baseline measures battery (US $20), post-intervention measures battery (US $20), and interview (US $25).

### Measures

#### Sociodemographic and medical variables

The following sociodemographic information will be collected via a brief background/demographic questionnaire and medical chart abstraction: participant age, sex assigned at birth, gender identity, race, and ethnicity. We will also collect data on participants’ current residence’s distance from SCH and whether they relocated to the Seattle area for treatment. The following disease- and treatment-related variables will be collected via medical chart abstraction: diagnosis, age at diagnosis, months/years since diagnosis, active treatment vs off treatment, months/years since treatment completion, and type(s) of treatment received (e.g., chemotherapy, radiation, surgery, HSCT).

#### Feasibility (Aim 1)

Feasibility will be assessed by the number of patients enrolled, session attendance, and attrition. Feasibility will be defined as ≥ 5 AYAs enrolled in each group offered, attendance ≥ 60% of sessions, and < 25% attrition (i.e., number of AYAs who withdraw from group). Reasons for dropout will be noted. Evidence indicates that ideal group size for group interventions depends on the target population, but that five or more members allow for meaningful relationships to form and for group cohesion to develop [29]. Our target numbers for feasibility are also informed by prior clinical experience and reflect the reality that some group members are on active treatment and have medical events that may interfere with group participation at times. We take these metrics into consideration when defining feasibility of the intervention given the unique challenges and needs of this population. We also anticipate that AYAs who are only able to attend 60% of sessions may still find the intervention to be acceptable/satisfactory. For patients who were referred to group but did not enroll, the reason for not enrolling (e.g., no longer interested, schedule conflict) will be noted in the referral in the electronic medical record when the scheduling team contacts the patient/family to enroll in the group.

#### Acceptability (Aim 1)

Acceptability of the telehealth group intervention will be assessed by an internally developed measure used widely within SCH to evaluate group-based interventions as part of routine measurement-based care. It was adapted from the Client Satisfaction Questionnaire (CSQ), which has excellent internal consistency and solid psychometric properties [30]. The CSQ has also been used among cancer populations, including adolescent and young adult cancer survivors [31, 32]. All changes made to the CSQ were to reduce respondent burden and tailor questions to specific clinical programs. This questionnaire was further adapted and modified for relevance to the High School Cancer Group and AYA Cancer Group. There are 16 items that assess group acceptability (i.e., group satisfaction) and telehealth acceptability. A threshold of 80% for acceptability ratings will be used as an indicator of intervention acceptability. This measure takes less than 10 min to complete.

#### Semi-structured patient interviews (Aim 2)

A semi-structured interview was developed for this study. Patient interviews will assess perceived benefits (including potential impact on social isolation and connectedness, sense of community, and psychological well-being), barriers/challenges, telehealth group acceptability, and suggestions for intervention refinement. It is anticipated that interviews will last approximately 30–45 min. Study staff will be trained by the first author (S. C.) and complete mock interviews prior to conducting interviews with participants. A copy of the interview guide is available from the first author upon reasonable request.

#### Patient-reported outcomes (Aim 3)

##### Revised UCLA Loneliness Scale

Participants will complete the Revised UCLA Loneliness Scale [33]. This 20-item self-report measure assesses social isolation (or lack of social connectedness). Sample items include the following: “I feel left out,” “I feel isolated from others,” “I have a lot in common with the people around me,” “There are people who really understand me,” and “There are people I can talk to.” Items are rated on a 4-point Likert scale (1 = never, 4 = often). Certain items are reverse scored. This widely used measure has demonstrated validity and reliability among adolescents and young adults.

##### *PROMIS* Depression Short Forms

Participants will complete the *PROMIS* Emotional Distress — Depression Short Form [34] (ages 18 years and above) or the *PROMIS* Pediatric Depression Short Form [35] (ages < 18 years). This 8-item self-report questionnaire assesses depressive symptoms in the past 7 days. Sample items on the adult form include the following: “I felt worthless,” “I felt helpless,” and “I felt depressed.” Sample items on the pediatric form include the following: “I felt sad,” “I was less interested in doing things I usually enjoy,” and “It was hard for me to have fun.” Items are rated on a 5-point Likert scale (1 = never, 5 = always). This measure takes approximately 2 min to complete. These *PROMIS* measures have demonstrated validity and reliability [34–36].

##### Benefit Finding/Burden Scale for Children

Participants will complete the Benefit Finding/Burden Scale for Children [37, 38]. This 20-item self-report measure assesses perceived benefit and burden associated with a specified event. For this study, we will examine the 10-item Benefit Finding subscale, which assesses one’s perception of personal growth resulting from an identified event. Sample items include the following: Having my illness… “has helped me become a stronger person,” “has helped me learn to deal better with my problems,” and “has taught me what is really important in life.” Items are rated on a 5-point Likert scale (“Not at all true for me” to “Very true for me”). This is a well-validated measure with excellent internal reliability that has been used in studies of children, adolescents, and young adults with cancer [39, 40].

### Analytic approach

Missing data will be examined and handled following best practices depending on the amount of missing data and patterns of missingness (e.g., pairwise deletion). We will include descriptives of patient demographic and disease characteristics to examine whether there are any obvious commonalities in dropout and to directly query and catalog reasons for dropout when possible. Data from participants in the AYA Cancer Group and the High School Group will be combined and analyzed together.

Data will be summarized using descriptive statistics in SPSS. For Aim 1, to assess feasibility and acceptability, the following metrics and descriptive statistics will be calculated: number of AYAs enrolled in each group (target: ≥ 5 AYAs per group), number of sessions attended (target: ≥ 60% of sessions), and attrition (target: < 25%). The number of AYAs referred to group who did not enroll and frequencies of their reasons for not enrolling will also be calculated. Acceptability ratings will be calculated with a target of 80% as an indicator of intervention acceptability.

For Aim 2, we will conduct a qualitative content analysis of interview data to assess patient experience and feedback in Dedoose, a web application for analyzing qualitative and mixed methods research (www.dedoose.com). We will develop an a priori codebook based on questions from the interview guide. At least two independent coders will code interview transcripts, and discrepancies will be resolved through discussion to reach consensus utilizing constant comparative methods [41, 42].

For exploratory Aim 3, scores for social isolation/social connectedness, depressive symptoms, and benefit-finding pre- and post-intervention will be computed, and simple descriptive statistics will be calculated. Because this is a feasibility pilot study that is not powered to detect significant outcomes, we will not conduct formal directional hypothesis testing [43, 44]. However, we plan to conduct paired samples *t*-tests to evaluate changes in scores from pre- to post-intervention in an exploratory manner. Exploration of preliminary efficacy will inform sample size and power of future trials.

## Discussion

Adolescents and young adults with cancer have unique psychosocial needs, and their opportunities to connect with peers with cancer are limited. Telehealth group interventions for AYAs with cancer are emerging in clinical settings in an effort to meet this critical need but remain under-researched. The goal of this pilot study is to examine the feasibility and acceptability of a telehealth peer support group intervention for AYAs with cancer. Findings from this study have the potential to impact psychosocial care of AYA oncology patients and programs and inform implementation of evidence-based care consistent with the Standards for Psychosocial Care for Children with Cancer and Their Families.

This study has several notable strengths, including important research and clinical innovations. Psychosocial interventions have moved to virtual platforms after the COVID-19 pandemic, and although this modality for care delivery is increasingly used, it remains largely untested. There is a wide research-practice gap in which the scientific evidence for telehealth interventions lags far behind its adoption in clinical practice [45]. This innovative project is timely in addressing an important gap in the literature, especially among AYAs with cancer, a unique and under-researched population. Additionally, this study employs a mixed-methods design [46, 47]. It centers AYAs’ voices by including patient stakeholder interviews and includes quantitative measures which have been limited in the extant research [4].

The group intervention itself has several noteworthy clinical innovations, including use of patient narratives and peer support. The model is patient centered in that discussion topics are prioritized by the patients in attendance, and group attendance is flexible to accommodate patient location (e.g., patients can join remotely from home, an infusion chair, or the inpatient unit) and sessions missed due to conflicting appointments or illness. The group is designed to be inclusive by including patients both on and off treatment rather than survivors only and patients with a heterogeneity of medical diagnoses. Importantly, separate high school and AYA groups address the diversity of experiences among AYAs and attend to specific developmental needs for patients across the AYA age spectrum.

This study has several limitations as well as potential practical and operational challenges and considerations. First, this is a small, single-site pilot study without a comparison group, and generalizability of findings for a single-site study is limited. Recruiting a diverse sample may also be challenging. Nonetheless, this study is an important first step and will serve as formative groundwork for future studies to test this intervention in a multi-site randomized controlled trial. Second, groups will include a heterogeneity of diagnoses and treatment phases; sample size will prohibit meaningful examination of potential differences across diagnoses and treatment phases. This will be important to examine in future larger studies. Diversity of experiences may be beneficial as this group intervention is intended to be broadly applicable to AYAs with cancer on and off treatment. Third, groups are limited to English-speaking participants. Active efforts are underway at our institution to obtain simultaneous interpretation for group interventions. Fourth, because AYAs self-select into groups, this may affect the representativeness of our sample, and this study carries risk of selection bias. We will not be able to collect data (e.g., feedback about barriers, reasons for nonparticipation) for AYAs who are not interested or referred to the group. This is another important future direction for research. We will track reasons for nonparticipation for AYAs who are referred to group but decline/do not enroll. Additionally, patients’ lack of awareness of availability of behavioral health services is often a barrier to receiving services. Some patients and families may not be aware of groups (e.g., patients who are seen less frequently at SCH, are further out from treatment, or who may be viewed by providers as less likely to want to participate in a group) precluding their participation, which may also affect representativeness of participants. Strong efforts will be made to increase patient and family awareness of the availability of the groups. Fifth, some group members have enrolled in prior cycles of group and are considered “repeaters,” which may also contribute to selection bias. As such, their baseline is not a true baseline. We will take this into consideration as we analyze the data. This observation suggests that some AYAs find continued benefit from the intervention prompting them to repeat group participation, which has interesting and important clinical implications. Finally, use of the adapted internally developed measure of acceptability was a pragmatic decision and includes items that assess both intervention acceptability and satisfaction. A future direction is to more clearly measure these constructs separately, such as by adapting the theoretical framework of acceptability questionnaire to assess acceptability of our telehealth group intervention for AYAs with cancer at the current and other treatment centers [48].

The next step in this program of research is to use findings on feasibility and acceptability to inform and make modifications to the intervention and refine a group curriculum that may serve as an exemplary model of care for increasing patient access to psychosocial standards of care for social interaction and psychosocial interventions at pediatric oncology sites. Future goals include evaluating the intervention’s effectiveness in an adequately powered randomized controlled trial. Development and testing of an evidence-based telehealth peer support group intervention for AYAs with cancer that improves psychosocial outcomes will meet a significant need for this vulnerable population.

## Data Availability

De-identified data will be available upon reasonable request from the corresponding author.

## Abbreviations

AYAs: Adolescents and young adults
HSCT: Hematopoietic stem cell transplant
SCH: Seattle Children’s Hospital
